# Surfactant-Assisted Distal Pulmonary Distribution of Budesonide Revealed by Mass Spectrometry Imaging

**DOI:** 10.3390/pharmaceutics13060868

**Published:** 2021-06-12

**Authors:** Riccardo Zecchi, Pietro Franceschi, Laura Tigli, Barbara Pioselli, Valentina Mileo, Xabier Murgia, Fabrizio Salomone, Giuseppe Pieraccini, Haruo Usada, Augusto F. Schmidt, Noah H. Hillman, Matthew W. Kemp, Alan H. Jobe

**Affiliations:** 1 Mass Spectrometry Center (CISM), Department of Health Sciences, University of Florence, Viale G. Pieraccini 6, 50139 Florence, Italy; riccardo.zecchi@unifi.it (R.Z.); giuseppe.pieraccini@unifi.it (G.P.); 2 Unit of Computational Biology, Research and Innovation Centre, Fondazione Edmund Mach via E. Mach, 1, 38010 San Michele all’Adige, Italy; pietro.franceschi@fmach.it; 3 Preclinical R&D, Chiesi Farmaceutici, Via Palermo, 26 A, 43122 Parma, Italy; L.Tigli@chiesi.com (L.T.); B.Pioselli@chiesi.com (B.P.); V.Mileo@chiesi.com (V.M.); 4 GAIKER Technology Centre, Department of Biotechnology, 48170 Zamudio, Spain; xabi_murgia@hotmail.com; 5 Centre for Perinatal and Neonatal Research, Tohoku University Hospital, Sendai 980-8575, Japan; haruo.usuda@uwa.edu.au; 6 Division of Obstetrics and Gynecology, University of Western Australia, Perth, WA 6009, Australia; matthew.kemp@uwa.edu.au (M.W.K.); alan.jobe@cchmc.org (A.H.J.); 7 Department of Pediatrics, University of Miami Miller School of Medicine, Miami, FL 33136, USA; augusto_schmidt@hotmail.com; 8 Division of Neonatology, Cardinal Glennon Children’s Hospital, Saint Louis University, Saint Louis, MO 63104, USA; Noah.Hillman@health.slu.edu; 9 Department of Obstetrics and Gynecology, Yong Loo Lin School of Medicine, National University of Singapore, Singapore 119228, Singapore; 10 Division of Neonatology and Pulmonary Biology, Cincinnati Children’s Hospital, School of Medicine, University of Cincinnati, Cincinnati, OH 45220, USA

**Keywords:** budesonide, Poractant alfa, mass spectrometry imaging, bronchopulmonary dysplasia, neonatal respiratory distress syndrome, premature lambs

## Abstract

Direct lung administration of budesonide in combination with surfactant reduces the incidence of bronchopulmonary dysplasia. Although the therapy is currently undergoing clinical development, the lung distribution of budesonide throughout the premature neonatal lung has not yet been investigated. Here, we applied mass spectrometry imaging (MSI) to investigate the surfactant-assisted distal lung distribution of budesonide. Unlabeled budesonide was either delivered using saline as a vehicle (*n* = 5) or in combination with a standard dose of the porcine surfactant Poractant alfa (*n* = 5). These lambs were ventilated for one minute, and then the lungs were extracted for MSI analysis. Another group of lambs (*n* = 5) received the combination of budesonide and Poractant alfa, followed by two hours of mechanical ventilation. MSI enabled the label-free detection and visualization of both budesonide and the essential constituent of Poractant alfa, the porcine surfactant protein C (SP-C). 2D ion intensity images revealed a non-uniform distribution of budesonide with saline, which appeared clustered in clumps. In contrast, the combination therapy showed a more homogeneous distribution of budesonide throughout the sample, with more budesonide distributed towards the lung periphery. We found similar distribution patterns for the SP-C and budesonide in consecutive lung tissue sections, indicating that budesonide was transported across the lungs associated with the exogenous surfactant. After two hours of mechanical ventilation, the budesonide intensity signal in the 2D ion intensity maps dropped dramatically, suggesting a rapid lung clearance and highlighting the relevance of achieving a uniform surfactant-assisted lung distribution of budesonide early after delivery to maximize the anti-inflammatory and maturational effects throughout the lung.

## 1. Introduction

Bronchopulmonary dysplasia (BPD) is a chronic lung disease affecting premature neonates born at low gestational ages (GA). BPD is associated with an arrested lung parenchymal and vascular development and often leads to long-term pulmonary sequelae [1]. The pathogenesis of BPD is complex and has not yet been entirely understood. However, lung inflammation is a hallmark of the disease [2], which may be triggered by several perinatal factors, including antenatal and postnatal infections and intensive therapies such as supplemental oxygen and invasive mechanical ventilation [3]. Although the iatrogenic effects of oxygen therapy and endotracheal ventilation are well known, they are of great therapeutic value for babies with severe surfactant deficiency developing the neonatal Respiratory Distress Syndrome (RDS) [4], and especially, for babies who have a poor respiratory drive at birth and cannot be adequately supported with more gentle treatments such as noninvasive ventilation.

Direct lung administration of budesonide has been proven to be an effective therapy for treating premature neonates at high risk of developing BPD [5,6,7]. Budesonide is a nonhalogenated corticosteroid with a high affinity for the glucocorticoid receptor that has widespread anti-inflammatory effects on several cell types, including macrophages, neutrophils, mast cells, and lymphocytes [8]. Lung delivery of budesonide also has maturational effects in premature animals, enhancing the expression of genes encoding surfactant proteins (SP) and the epithelial sodium channel (ENaC) [9,10]. The NEUROSIS trial demonstrated that early inhaled budesonide delivered with a pressurized metered-dose inhaler (pMDI) reduced the incidence of BPD in babies with a GA ranging between 23 and 27 weeks [5]. Nonetheless, the mortality in the intervention group was slightly higher than in the placebo group. Budesonide lung deposition was not reported, but it could be expected to be relatively low given the poor aerosol delivery efficiency in premature babies [11]. Yeh et al. envisaged an alternative budesonide administration method for very-low-birth-weight babies (mean GA 26.5 weeks) requiring mechanical ventilation and oxygen supplementation. The method consists of a combination therapy of budesonide (0.25 mg/kg) mixed with 100 mg/kg of surfactant (Beractant, Survanta^®^, Abbott Laboratories, Abbott Park, IL, USA) delivered as an intratracheal bolus [6,12]. This delivery strategy guarantees a high initial lung bioavailability of budesonide and takes advantage of the good spreading properties of surfactant to boost budesonide’s lung distribution. Remarkably, the group of babies randomized to receive the combination therapy had lower interleukin levels in their tracheal aspirates, received fewer surfactant treatments, and, most importantly, registered a significantly lower incidence of BPD or death than babies treated with surfactant alone [6].

The pulmonary distribution of budesonide delivered in combination with surfactant has been investigated using fluorescence imaging in mice [13] and radiolabeled budesonide in rats and adult rabbits [14,15]. The studies performed with healthy rodents demonstrated an improved budesonide distribution in combination with surfactant [13,14]. In contrast, the study by Fajardo et al. conducted in adult rabbits did not reveal differences in the peripheral distribution of budesonide using either surfactant or saline as vehicles [15]. Of note, these studies were exclusively performed with the bovine surfactant Beractant and using adult animal models with a healthy pulmonary status in most cases. Therefore, the lung distribution of budesonide using other commercially available surfactant preparations and/or premature animal models with a primary surfactant deficiency has not been yet investigated. Therefore, the present study was designed to address the lung distribution of budesonide in premature lambs either delivered alone, using saline as a vehicle, or in combination with a standard dose of Poractant alfa (Curosurf^®^, Chiesi Farmaceutici, Parma, Italy). We applied mass spectrometry imaging (MSI) to visualize the budesonide distribution in transversal sections of the right lower lung lobes of premature lambs. MSI enabled the label-free detection and mapping of budesonide and the essential constituent of Poractant alfa, the porcine SP-C. Additionally, we applied spatial statistics to the analysis of the signal intensity of budesonide to further confirm the superior surfactant-assisted distal pulmonary distribution of budesonide.

## 2. Materials and Methods

### 2.1. Chemicals and Materials

Ethanol, methanol, trifluoroacetic acid (TFA), Girard’s reagent P (GirP), budesonide chemical standard, and MALDI matrices 2,5-dihydroxybenzoic acid (DHB) and ferulic acid (FA) were purchased from Sigma-Aldrich Italy (Milan, Italy). Distilled water was taken from a MilliQ apparatus (Millipore Merck, Milan, Italy). Histology glass slides were model Superfrost Plus (Thermo Scientific, Waltham, MA, USA), and ITO slides were purchased from Bruker Daltonik (Bremen, Germany). Meyer’s hematoxylin, eosin alcoholic solutions for tissue staining, and xylene-based tissue fixing glue were purchased from Diapath (Milan, Italy). Poractant alfa (Curosurf^®^, 80 mg of phospholipids/mL) was provided by Chiesi Farmaceutici (Parma, Italy), and budesonide (Pulmicort, 0.5 mg/mL) was obtained from AstraZeneca (Wilmington, DE, USA).

### 2.2. Animal Experiments

All animal experiments were approved by the animal welfare committee of the University of Western Australia (RA/3/100/1511, 07 May 2018). Merino sheep were time-mated to yield lambs of 126–127 days’ GA (term 150 days). Lambs were surgically delivered as previously described [10] and assigned to one of the experimental groups: (1) Lambs in the surfactant + budesonide group (SF+BUD 1′, *n* = 5) received an intratracheal bolus of Poractant alfa (200 mg/kg, 2.5 mL/kg) gently mixed with 0.25 mg/kg of budesonide. A 3 kg birth weight was used as an estimate for surfactant and budesonide dosing of all animals. (2) Lambs in the BUD 1′ group (*n* = 5) received an intratracheal dose of 0.25 mg/kg of budesonide; the volume of the intratracheal bolus was adjusted with saline to match the instillation volume of the SF+BUD 1′ group. Lambs allocated to SF+BUD 1′ and BUD 1′ groups were mechanically ventilated for just 1 min after receiving the intratracheal bolus. Mechanical ventilation was provided with a Fabian ventilator (Acutronic, Bubicon, Switzerland) with a peak inspiratory pressure (PIP) of 30 cmH_2_O, a positive end-expiratory pressure of 5 cmH_2_O, a rate of 50 breaths/min, an inspiratory time of 0.5 s, with 40% heated and humidified oxygen. (3) Lambs in the SF+BUD 120′ group (*n* = 5) received an intratracheal bolus of Poractant alfa (200 mg/kg, 2.5 mL/kg) gently mixed with 0.25 mg/kg of budesonide and were ventilated for 120 min. In these lambs, the PIP was adjusted not to exceed a tidal volume of 8 mL/kg. Two additional lambs received 200 mg/kg of Poractant alfa but never received budesonide; they were ventilated for 6 h and were included in the study as negative controls for the MSI budesonide detection.

At necropsy, lungs were removed, visually inspected, and separated into right and left lungs. The right lung was inflated statically with air to 35 cmH_2_O and suspended in a Dewar flask containing liquid nitrogen in the bottom. Distending pressure was then decreased to 20 cmH_2_O and the lung froze above the liquid nitrogen in about 15 min. The lower lobes of the right lungs were delivered frozen on dry ice to the University of Florence, where they were stored at −80 °C until MSI analysis.

### 2.3. Tissue Sectioning

**Figure 1:**
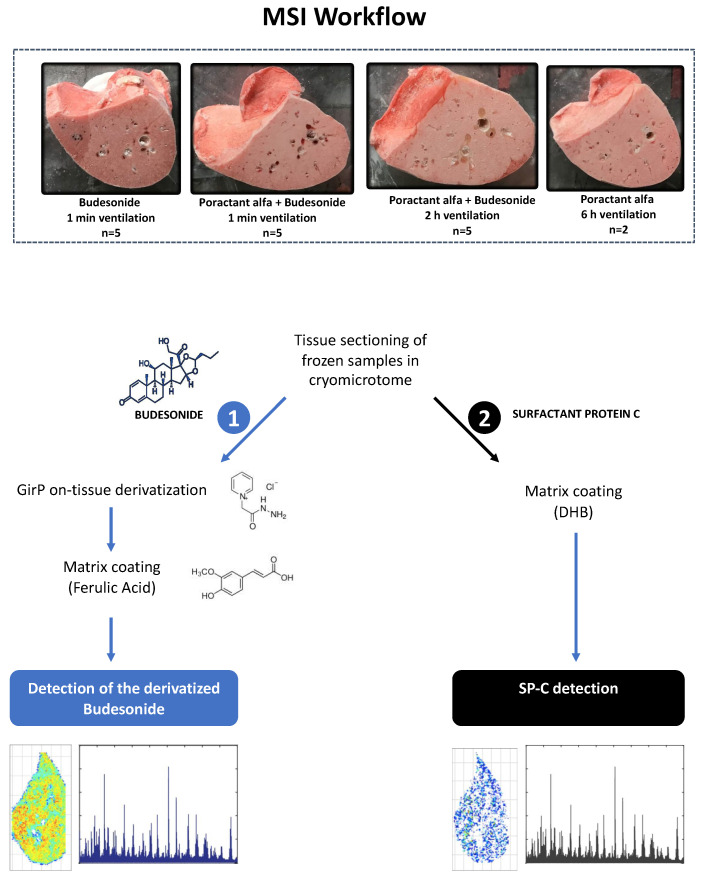
Mass spectrometry imaging (MSI) workflow applied in the present study. The lower lobes of right lungs from premature lambs receiving either budesonide alone or in combination with surfactant were analyzed. Two additional animals that did not receive budesonide were also included in the study as negative controls. Two consecutive transverse tissue sections of 20 μm thickness were obtained from each sample. (1) One of the sections was used for budesonide analysis, which required on-tissue derivatization with Girard reagent P (GirP) followed by matrix coating with ferulic acid. (2) The second tissue section was used to analyze the spatial distribution of surfactant protein C (SP-C). A MALDI-LTQ-Orbitrap XL mass spectrometer was used for budesonide analysis, whereas an Ultraflex III TOF/TOF mass spectrometer was used for SP-C analysis. Data processing and 2D ion intensity maps for each analyte were generated with “R” software (Version 4.1.0. R Core Team (2021). R: A language and environment for statistical computing. R Foundation for Statistical Computing, Vienna, Austria. URL https://www.R-project.org/, accessed on 11 June 2021).

The cryostat CM1860UV (Leica Microsystems, Wetzlar, Germany) was used for sectioning the lung lobes as fresh frozen tissue. The lobes were cut among transversal plane at middle height obtaining tissue section of approximately 50 × 20 mm. Two consecutive sections were cut at 20 µm thickness: one section was thaw-mounted over a Superfrost slide for budesonide analysis and the second one was thaw-mounted over an ITO slide for SP-C analysis (Figure 1). All the prepared glass slides were posed in a vacuum desiccator (15 min) and allowed to dry prior further phases of sample preparation.

### 2.4. Sample Preparation and MALDI-MSI Analysis for Budesonide Detection

Budesonide is challenging to detect by MSI as it is not easily protonated or deprotonated. Therefore, to improve the detection of budesonide in lung tissue sections, an on-tissue chemical derivatization protocol was adopted as recently described by Zecchi et al. [16]. Briefly, this approach uses GirP as an on tissue derivatizing agent, followed by FA matrix coating. After coating, samples were finally dried in a vacuum desiccator for 15 min. Acquisition was performed on a vacuum MALDI-LTQ-Orbitrap XL mass spectrometer (Thermo Fisher, San Josè, CA, USA) using a raster size of 400 × 400 µm in the mass range from *m*/*z* 185 to 650 in full scan positive ion mode. 2D ion intensity maps were created by plotting the intensity of budesonide-GirP molecular ion (564.308 *m*/*z*). Signal normalization was performed by using the derivatized triamcinolone ion (568.283 *m*/*z*). All ion traces were extracted with a tolerance of 0.005 Da.

### 2.5. Sample Preparation and MALDI-MSI Analysis for SP-C Detection

The matrix (DHB, 30 mg/mL in 50% ethanol with 0.2% TFA) was applied in 30 cycles using IMatrix Spray device (density of 0.8 µL/cm^2^, nitrogen flow at 120 bar, needle distance of 6 cm, heated beds at 60 °C). After matrix coating, samples were dried in a vacuum desiccator for 15 min. MALDI-MSI analyses were conducted on an Ultraflex III TOF/TOF mass spectrometer (Bruker Daltonik, Bremen, Germany). The instrument worked in positive ion reflectron mode with full scan acquisition in the range of 2000 to 5000 *m*/*z*. Spectra were acquired by rastering the section surface with a step of 400 µm in both x and y directions. 2D ion intensity maps were created by plotting the intensity of porcine SP-C molecular ion (4188 *m*/*z*). All ion traces were extracted with a tolerance of 1 Da.

For both budesonide and SP-C, data were acquired as centroided and then converted with “RAW to imzML” software (Giessen University, Giessen, Germany). Data management and visualization were performed in *R* (Version 4.1.0. R Core Team (2021). R: A language and environment for statistical computing. R Foundation for Statistical Computing, Vienna, Austria), relying on the following libraries: *tidyverse*, *grid*, and *egg* for data visualization and manipulation [17,18], and *gstat*, *rgdal, rgeos*, *igraph*, and *sp* for spatial statistics [19,20,21,22].

### 2.6. Spatial Statistics

The signal of each tissue section was first scaled and mean-centered, and the intensity was binned in quartiles. The pixels in the 2nd and 4th quartile of the budesonide intensity signal were then extracted from the dataset and plotted as 2D ion intensity maps for the lungs of animals in the SF+BUD 1′ and BUD 1′ groups. The physical distance of each high-intensity pixel (4th quartile) from the tissue border was determined for each animal. Tissue borders were delineated by automatically estimating the borders of the spatial area where *heme* signal was present by using a piecewise linear curve (“alpha hull”) [23]. The *heme* signal (616.176 *m*/*z*) is indeed detected only in correspondence with the presence of tissue.

Adjacent high-intensity pixels within the lung tissue sections were defined as budesonide “clumps”; the number and the median size (in pixels) of the clumps were calculated for each animal, and comparisons between SF+BUD 1′ and BUD 1′ groups were performed applying a nonparametric Wilcoxon rank sum test. A *p* < 0.05 was accepted as significant.

## 3. Results

### 3.1. Animal Characteristics

The lamb characteristics are summarized in Table 1. There were no differences in the male-to-female ratio or in the GA of the animals, although a marginally significant difference was found in the birthweight comparisons (*p* = 0.027, one-way ANOVA) with animals in the SF+BUD 1′ having a lower mean birth weight. This was unexpected as lambs were delivered at the same GA. Post hoc analysis revealed a significant difference between SF+BUD 1′ and BUD 1′ groups, but no between SF+BUD 1′ and SF+BUD 120′ groups.

### 3.2. Budesonide Detection in Transverse Tissue Sections of the Right Lower Lobe

**Figure 2:**
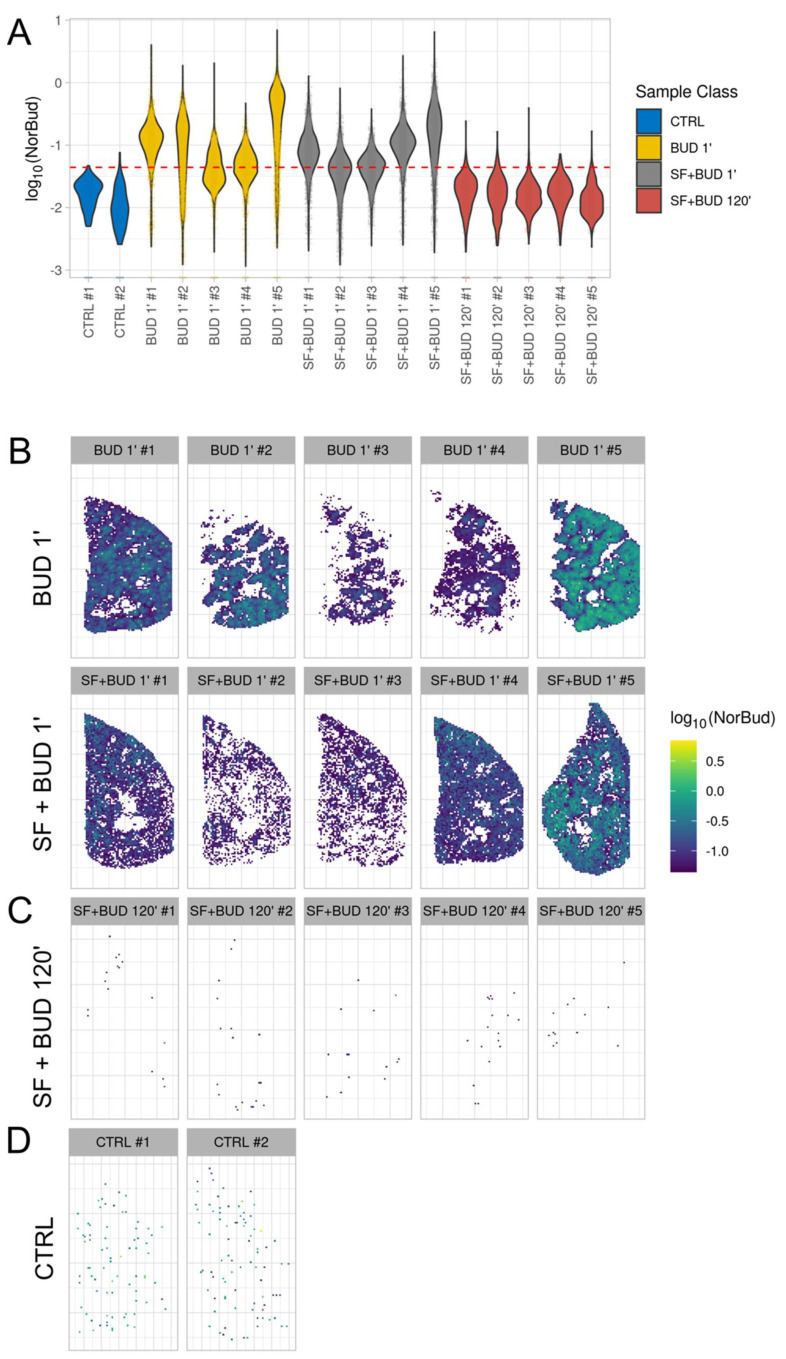
Analysis of budesonide lung distribution by MSI. (**A**) Violin plots displaying the logarithm of the intensity of each animal included in the study. The dashed red line represents the signal density threshold for budesonide, which was set by taking the control animals (never received budesonide) as a reference. (**B**) 2D ion intensity images displaying the distal budesonide lung distribution in samples obtained from premature lambs that were ventilated for just 1 min after either receiving intratracheal budesonide (0.25 mg/kg, top row, BUD 1′) or budesonide (0.25 mg/kg) combined with surfactant (200 mg/kg, bottom row, SF+BUD 1′). (**C**) 2D ion intensity images displaying the distal budesonide lung distribution in samples obtained from premature lambs that were ventilated for 120 min after receiving budesonide (0.25 mg/kg) combined with surfactant (200 mg/kg, SF+BUD 120′). (**D**) 2D ion intensity images of untreated control (CTRL) animals, which were used to set the budesonide intensity signal threshold.

The logarithms of the budesonide signal’s intensity of all the animals included in the study are shown in Figure 2A. The “violin” plot shows the density of the pixels along the intensity axis. First, the intensity signals of the control animals that never received budesonide were used to set an intensity threshold. Therefore, the budesonide signal above the threshold was used to implement 2D ion intensity images of the tissue sections. Budesonide was detected in the distal right lower lung lobes of all animals extracted after just 1 min of mechanical ventilation (Figure 2B). However, the budesonide distribution displayed a patchier pattern in the lung sections of the BUD 1′ group, particularly in three out of five tissue sections. Conversely, whenever budesonide was delivered in combination with surfactant, a more homogeneous distribution of the intensity signal was observed, covering a higher lung tissue area and clearly defining the borders of all tissue sections of the SF+BUD 1′ group.

The budesonide intensity in the lung tissue sections of the animals ventilated for 2 h was dramatically reduced, presumably by clearance from the lungs, as denoted by the anecdotal number of pixels displaying an intensity signal above the threshold (Figure 2C).

### 3.3. Comparison of the Lung Localization of Budesonide and SP-C

**Figure 3:**
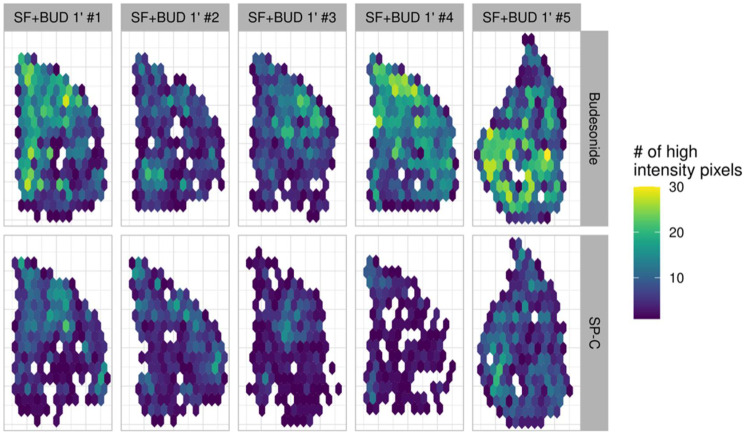
Two-dimensional density maps of the budesonide (top row) and SP-C (bottom row) from premature lambs that were ventilated for just. 1 min after either receiving intratracheal budesonide (0.25 mg/kg) combined with surfactant (200 mg/kg, SF+BUD 1′). SP-C and budesonide were analyzed in consecutive lung tissue sections using different sample preparation protocols.

To investigate the association in the distribution between surfactant and budesonide, the intensity signals of budesonide and SP-C were mapped in two consecutive tissue sections of the right lower lung lobes (Figure 3). The intensity signal of SP-C was markedly lower compared to the signal of budesonide. This could be expected as SP-C is a qualitatively very relevant but numerically minor component of the exogenous surfactant Poractant alfa (~0.7 *w*/*w*%) [24]. Nevertheless, the areas with a more intense signal of SP-C appear to be correlated with the areas of higher budesonide intensity, suggesting that budesonide may travel associated with surfactant along the lung parenchyma.

### 3.4. Statistical Analysis of Budesonide Distribution

**Figure 4:**
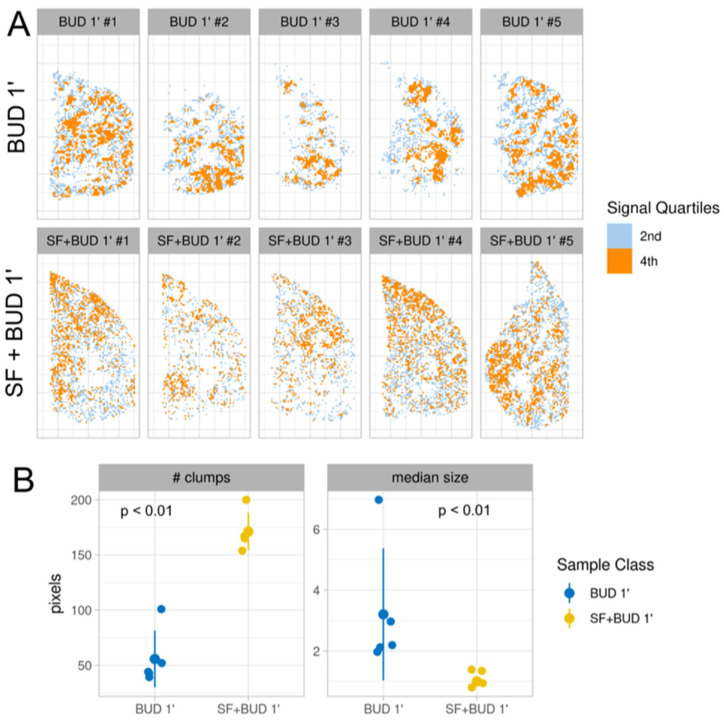
Statistical analysis of budesonide distribution. (**A**) 2D ion intensity images displaying the distribution of the 2nd (grey) and 4th (orange) quartiles of the budesonide intensity. The images were obtained from distal right lung samples from premature lambs that were ventilated for just 1 min after either receiving intratracheal budesonide (0.25 mg/kg, top row, BUD 1 min) or budesonide (0.25 mg/kg) combined with surfactant (200 mg/kg, bottom row, SF+BUD 1 min). (**B**) Comparison of the number of clumps quantified in the ion intensity images and their median size. *p* < 0.01, Wilcoxon rank sum exact test.

The qualitative differences observed in budesonide distribution between BUD 1′ and SF+BUD 1′ groups were further investigated through an extended statistical analysis. For that purpose, the signal of each section was first scaled and mean-centered, and the intensity was binned in quartiles. The pixels within the second and fourth intensity quartiles of the budesonide signal were mapped independently across the lung samples, as shown in Figure 4A. The resulting images reveal a different distribution pattern between groups. The fourth quartile (high-intensity pixels) signal appears as aggregated pixels or high-intensity clumps in the animals in the BUD 1′ group, which show a diffuse distribution across the sample. Conversely, high-intensity pixels appear better distributed across the tissue sections in the SF+BUD 1′ group animals in smaller clumps and fairly well distributed towards the sample periphery. A quantitative comparison of the mean number of clumps (i.e., interconnected pixels in the fourth quartile intensity) between groups revealed a significantly higher number of clumps with a lower median pixel size in the SF+BUD 1′ group (*p* < 0.01, Figure 4B).

**Figure 5:**
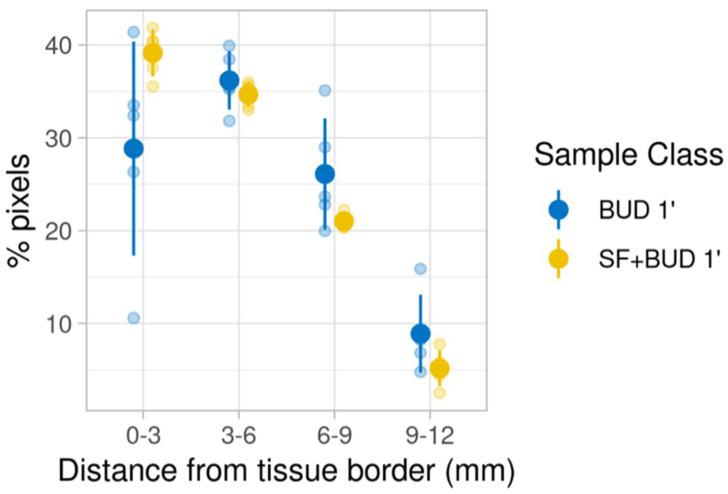
Distribution of the pixels belonging to the 4th intensity quartile of the budesonide signal as a function of their distance from the tissue border. In blue, data from lambs receiving intratracheal budesonide alone (0.25 mg/kg); in yellow, data from lambs receiving budesonide (0.25 mg/kg) combined with surfactant (200 mg/kg). No statistical differences were noted between groups; at 0–3 mm from the tissue border, *p* = 0.09; at 3–6 mm, *p* = 0.6; at 6–9 mm, *p* = 0.15; at 9–12 mm, *p* = 0.09; Wilcoxon rank sum test.

Spatial statistics were also performed to investigate the peripheral distribution of budesonide. The tissue border of each sample was delineated using the “*heme*” signal, which is a characteristic signal of the tissue. Then, the distribution of the physical distance of each high-intensity pixel from the tissue border was determined (Figure 5). Compared with the BUD 1′ group, animals in the SF+BUD 1′ group had more high-intensity pixels near the tissue border, indicative of a higher amount of budesonide reaching peripheral lung areas. However, the differences between groups were not statistically different at a certain distance from the tissue border.

## 4. Discussion

The combination therapy with intratracheal surfactant plus budesonide is currently undergoing clinical development [25,26,27]. However, the potent anti-inflammatory properties of corticosteroids for the treatment and prevention of BPD have been previously explored in various clinical trials. For instance, intravenous dexamethasone significantly reduces the rate of BPD in premature infants [28], but it has been associated with long-term adverse neurologic disabilities [29]. Topical pulmonary delivery of corticosteroids was proposed as an administration method that would reduce the systemic exposure of the i.v. route. In this regard, Bassler et al. reported a lower incidence of BPD in babies with a GA of 23–28 weeks treated with early inhaled budesonide compared to placebo [5]. A follow-up study of the long-term effects of the treatment did not find adverse neurodevelopmental effects associated to the budesonide treatment, although mortality was higher in the intervention group [30]. The babies in the intervention group received a nominal dose of 0.4 mg of budesonide from a pMDI twice daily in the first 14 days, followed by 0.2 mg twice daily from day 15. The mean duration of the treatment was 34 days. Although the lung deposition of budesonide was not reported, the delivery efficiency via aerosolization must have been hampered by the low lung deposition rates reported in premature neonates when using pMDIs (less than 1% of the nominal dose) [31].

For those babies requiring mechanical ventilation and surfactant therapy for RDS management, Yeh et al. proposed the combined intratracheal delivery of budesonide (0.25 mg/kg) and surfactant (Beractant 100 mg/kg) [12]. The combination therapy was associated with a significant reduction in the incidence of BPD or death in premature babies [6]. Notably, 97.7% of the babies in the intervention group (128 out of 131) received just one (64.9%) or two (32.8%) doses of budesonide within the first 24 h of life. Recently, an observational study conducted in premature babies (≤1250 g) receiving surfactant plus budesonide after failing continuous positive airway pressure (CPAP) therapy has demonstrated less severe BPD, decreased mechanical ventilation use, and earlier discharge [7]. Therefore, compared with inhaled budesonide, the surfactant-assisted combination therapy significantly enhances the pulmonary delivery efficiency of budesonide. Nevertheless, this budesonide delivery method is limited to babies requiring surfactant therapy.

The use of exogenous surfactant as a vehicle for pulmonary drug delivery is not new and has been previously proposed for the co-delivery of other drugs such as antibiotics [32,33,34,35], adenoviral vectors [36], immunosuppressive drugs [37], antioxidants [38,39], and other anti-inflammatory molecules [40,41,42], including other types of corticosteroids (dexamethasone and beclomethasone) [43,44]. The main advantages of using surfactant as a vehicle are the high intrapulmonary bioavailability of the co-delivered drug (i.e., approximately 90% of the intratracheally delivered surfactant deposits in the lungs) [45] and the intrinsic ability of surfactant to guarantee a rapid adsorption and spreading along air-liquid interfaces towards the lung periphery [46].

The lung distribution of budesonide delivered in combination with surfactant has been investigated in a few preclinical studies with inconsistent results. Huang et al. indirectly investigated the pulmonary distribution of budesonide in healthy mice using a fluorescent tracer mixed with surfactant and budesonide [13]. The lung distribution of the fluorescent dye 15 min after the intratracheal injection was slightly enhanced if it was delivered in combination with surfactant and budesonide compared with the delivery of the dye with budesonide alone. Cheng et al. demonstrated an enhanced distribution of the radiolabeled ^18^F-budesonide towards the lung periphery of healthy rats that received the combination therapy; conversely, the animals receiving intratracheal budesonide alone displayed a central budesonide distribution with most of the radioactive signal concentrated near the trachea [14]. Fajardo et al. compared the distribution of ^3^H-budesonide using saline or surfactant as vehicles in healthy and surfactant-depleted adult rabbits [15]. The authors reported no differences in terms of lung distribution using saline or surfactant and found less budesonide in the peripheral lung areas compared with the small and large airways. These differences reported between studies may be due to the use of different methods to assess the budesonide distribution (fluorescent tracer vs. radioactive labeling) or by differences in the animal models. However, note that all these studies were conducted with Beractant, the same natural surfactant preparation used in the randomized clinical trial by Yeh et al. [6]. Moreover, they used adult and mostly healthy animal models, a feature that may have influenced the pulmonary distribution pattern of budesonide.

In this work, we investigated the distal distribution of budesonide combined with Poractant alfa using premature lambs with a primary surfactant deficiency. Compared with Beractant, Poractant alfa is presented at a higher phospholipid concentration (80 vs. 25 mg/kg) and is licensed to be administered either at 100 (1.25 mL/kg) or 200 mg/kg (2.5 mL/kg). Both surfactant preparations are effective in the treatment of RDS, although according to a large meta-analysis, Poractant alfa delivered at a dose of 200 mg/kg significantly reduces the mortality compared to Beractant, which is delivered at 100 mg/kg [47]. In vitro and in vivo studies have demonstrated the compatibility of Poractant alfa with the standard intratracheal budesonide dose of 0.25 mg/kg, showing a good stability of budesonide within the phospholipid suspension for 24 h, no detrimental effects in the surface tension reducing properties of Poractant alfa, and positive long-term effects on lung performance [48,49,50]. Moreover, the intratracheal delivery of Poractant alfa and budesonide reduces the concentration of proinflammatory cytokines in the bronchoalveolar lavage fluid and the expression of inflammatory markers in the lung, liver, and brain of preterm lambs exposed to various prenatal and postnatal insults, implying both a local and systemic effect of intratracheally delivered budesonide [10,51,52,53]. To complete the preclinical profiling of the combination of Poractant alfa and budesonide, we applied MSI to visualize the pulmonary distribution of budesonide delivered with or without Poractant alfa.

MSI is a mass spectrometry technique that provides label-free visualization of the analytes of interest. We have previously utilized MSI to investigate the pulmonary distribution of natural (Poractant alfa) and synthetic (CHF5633, Chiesi Farmaceutici, Parma, Italy) surfactants in surfactant-depleted rabbits and in premature lambs [54,55]. In these studies, we visualized the pulmonary distribution of exogenous surfactants by following the unique spectral signals of the porcine SP-C and synthetic SP-C analogue ions. Using this work as a starting point, our primary aim was to simultaneously locate SP-C and budesonide in the same lung tissue section. However, the poor ionization of budesonide in lung tissue prevented us from the application of this strategy. To increase the budesonide signal, an on-tissue derivatization step with the GirP reactant had to be performed, as recently described by Zecchi et al. [16], which required a dedicated sample preparation. Therefore, SP-C and budesonide images were obtained from consecutive tissue sections. Compared with the BUD 1′ group, the 2D ion intensity maps obtained for the SF+BUD 1′ group showed a more homogeneous distribution of budesonide throughout the samples. Besides, we found similar distribution patterns for the SP-C and budesonide intensity signals in the consecutive lung sections of the animals in the SF+BUD 1′ group, which indicates that budesonide was transported across the lungs to the lung periphery by the exogenous surfactant. The analysis of the 2nd and 4th quartiles of the intensity signal of budesonide revealed a nonuniform and rather patchy distribution of budesonide alone, which appeared clustered in clumps that were significantly lower numerically and bigger in size compared to the clumps observed in the SF+BUD 1′ group. Moreover, spatial statistics revealed more high-intensity pixels in the sample periphery of the animals allocated in the SF+BUD 1′ group, which supports the enhanced surfactant-assisted budesonide distribution. Notably, however, budesonide was also detected in the distal lung of the animals that did not receive surfactant, with one of the tissue sections in the BUD 1′ group displaying a fairly homogeneous budesonide distribution. We speculate that the delivery of budesonide to the fluid-filled lungs of freshly delivered lambs may have enhanced the pulmonary distribution of budesonide to the lower right lung lobe, even in the absence of surfactant.

Topically administered budesonide shows a rapid absorption half-life [8]. In premature infants receiving intratracheal budesonide in combination with Beractant, the mean plasma half-life was reported to be 4.13 h with a maximum plasma concentration (C_max_) of 20 ng/mL; the authors estimated that approximately 4% of the instilled budesonide was absorbed to the systemic circulation in the first 8 h following the intratracheal delivery [12]. This relatively low systemic absorption was attributed to a surfactant-enhanced budesonide uptake by lung cells, where budesonide undergoes a reversible conjugation with fatty acids and could be stored as budesonide esters [56]. This hypothesis was partially supported by the recent study by Kothe et al. who found a 40% increase of the budesonide lung levels if the lung tissue of premature lambs that received 0.25 mg/kg of budesonide was subjected to a de-esterification step before analysis [10]. Nevertheless, taken the unesterified and the esterified budesonide together, the overall budesonide levels in the lungs of preterm lambs were reduced by almost 93% two hours after intratracheal administration [10]. Our MSI results are in line with this finding and showed a dramatic drop of the budesonide intensity signal in the 2D ion intensity maps of the SF+BUD 120′ group.

The study has some limitations from the sample processing of MSI. Unfortunately, the poor ionization of budesonide did not allow analyzing the colocalization of budesonide and SP-C in the same lung tissue section, and therefore the association between the lung localization of both analytes had to be estimated from consecutive tissue slides from each animal. The lungs of the lambs are relatively big compared to other laboratory animals such as rodents, precluding the option of analyzing the whole lungs in a single MSI experiment. Indeed, these big tissue sections constrained us to adopt a spatial resolution of 400 × 400 µm in the analysis of each tissue slide, due to the low duty cycle of the mass analyzers (every sample processed with this analytical method required approximately 6–7 h). Therefore, instead of analyzing all lung lobes, we analyzed transverse sections of the right lower lung lobes of all animals, which provides a fair estimation of the distal pulmonary distribution of budesonide. The surfactant-assisted distal lung distribution of budesonide was investigated one min and two hours after therapy. Future studies should include repeated analysis (e.g., at 15 and 60 min) to characterize the budesonide distribution kinetics over time. Last, lambs received the same dose of budesonide based on an estimated birth weight of 3 kg. Consequently, the animals in the SF+BUD 1′ group received slightly more budesonide than animals in the BUD 1′ group.

## 5. Conclusions

This MSI study provides direct evidence of the relatively uniform, surfactant-assisted budesonide distribution in the distal lungs of premature lambs. MSI enabled the visualization of the differential distribution patterns of budesonide delivered with saline or in combination with Poractant alfa. The analysis of the 2nd and 4th intensity quartiles of the budesonide signal intensity revealed a patchy pulmonary distribution of budesonide delivered with saline; in contrast, if budesonide was delivered in combination with Poractant alfa, a more homogeneous distribution was observed, with budesonide being detected in the boundaries of the tissue section after just 1 min of ventilation. Two hours after the intratracheal delivery of the combination therapy, budesonide was almost fully cleared from the lungs, highlighting the relevance of achieving a uniform surfactant-assisted lung distribution of budesonide early after its intratracheal delivery to maximize a uniform anti-inflammatory and maturational effects of the therapy.

## Figures and Tables

**Table 1:** Characteristics of the lambs included in this study.

	BUD 1′	SF+BUD 1′	SF+BUD 120′
Birth weight (kg)	3.41 ± 0.21	2.73 ± 0.23	3.20 ± 0.52
Male:Female ratio	1:4	1:4	1:4
Gestational age (d)	126 ± 1	126 ± 1	126 ± 1

BUD, budesonide; SF, surfactant.

## Data Availability

Data will be available upon reasonable request.
